# Connexin Expression Is Altered in Liver Development of *Yotari* (*dab1 -/-*) Mice

**DOI:** 10.3390/ijms221910712

**Published:** 2021-10-02

**Authors:** Vlatka Paštar, Mirela Lozić, Nela Kelam, Natalija Filipović, Branka Bernard, Yu Katsuyama, Katarina Vukojević

**Affiliations:** 1 Mediterranean Institute for Life Sciences (MedILS), Meštrovićevo šetalište 45, 21000 Split, Croatia; vlatka.pastar@medils.hr (V.P.); branka.bernard@medils.hr (B.B.); 2 Department of Anatomy, Histology and Embryology, School of Medicine, University of Split, Šoltanska 2, 21000 Split, Croatia; mirela.lozic@mefst.hr (M.L.); nela.kelam@mefst.hr (N.K.); natalija.filipovic@mefst.hr (N.F.); 3 Department of Anatomy, Shiga University of Medical Science, Otsu 520-2192, Japan; kats@belle.shiga-med.ac.jp; 4 Department of Medical Genetics, School of Medicine, University of Mostar, 88000 Mostar, Bosnia and Herzegovina

**Keywords:** gap junction, liver development, Cx26, Cx32, Cx37, Cx40, Cx43, Cx45, Panx1, AIF, *yotari*

## Abstract

Disabled-1 (Dab1) protein is an intracellular adaptor of reelin signaling required for prenatal neuronal migration, as well as postnatal neurotransmission, memory formation and synaptic plasticity. *Yotari*, an autosomal recessive mutant of the mouse *Dab1* gene is recognizable by its premature death, unstable gait and tremor. Previous findings are mostly based on neuronal abnormalities caused by *Dab1* deficiency, but the role of the reelin signaling pathway in nonneuronal tissues and organs has not been studied until recently. Hepatocytes, the most abundant cells in the liver, communicate via gap junctions (GJ) are composed of connexins. Cell communication disruption in *yotari* mice was examined by analyzing the expression of connexins (Cxs): Cx26, Cx32, Cx37, Cx40, Cx43 and Cx45 during liver development at 13.5 and 15.5 gestation days (E13.5 and E15.5). Analyses were performed using immunohistochemistry and fluorescent microscopy, followed by quantification of area percentage covered by positive signal. Data are expressed as a mean ± SD and analyzed by one-way ANOVA. All Cxs examined displayed a significant decrease in *yotari* compared to wild type (wt) individuals at E13.5. Looking at E15.5 we have similar results with exception of Cx37 showing negligible expression in wt. Channels formation triggered by pathological stimuli, as well as propensity to apoptosis, was studied by measuring the expression of Pannexin1 (Panx1) and Apoptosis-inducing factor (AIF) through developmental stages mentioned above. An increase in Panx1 expression of E15.5 yotari mice, as well as a strong jump of AIF in both phases suggesting that yotari mice are more prone to apoptosis. Our results emphasize the importance of gap junction intercellular communication (GJIC) during liver development and their possible involvement in liver pathology and diagnostics where they can serve as potential biomarkers and drug targets.

## 1. Introduction

The liver is one of the first organs where the connexin proteins (Cxs) were described [1]. Since it is composed of parenchymal (hepatocytes) and nonparenchymal cells (Kupfer cells, sinusoidal endothelial cells, hepatic stellate cells (Ito cells), pit cells (natural killer cells) and biliary epithelial cells (cholangiocytes), understanding cell communication is indispensable [2,3]. Hepatocytes, the most abundant cells in the liver, communicate via gap junctions (GJ) which are formed by interactions of hemichannels (connexons) of neighboring cells, each consisting of six connexin proteins. Connexins are involved in cell growth, differentiation and death and nomenclature refers to their molecular weight expressed in kilodaltons [4]. Apart from connexins, which can form hemichannels and gap junctions, pannexins (Panxs) form only channels [5,6,7] taking part in cell inflammation and death processes [8,9,10]. It is a three-member family of proteins (Panx1, Panx2, Panx3) [11] similar to connexins by structure and permeability [7]. While connexin gap junctions control intercellular communication, connexin and pannexin (hemi)channels enable communication between the cell and extracellular environment [12]. Another difference is the fact that gap junctions are usually open in physiological conditions, while (hemi)channels open by several pathological triggers such as membrane depolarization increase, increase in intracellular calcium, decrease in extracellular calcium, mechanical stimulation, oxidative stress, changes in connexin phosphorylation, ischemia/reperfusion insults and inflammatory conditions [13]. Upon cell growth and differentiation, as well as in liver disease, the connexin pattern of expression is altered [14,15]. Hepatocytes mostly express Cx32 while Cx26 is present in a much lesser amount. Cx43 is expressed by nonparenchymal cells like Kupfer and stellate cells, while Cx37 and Cx40 are expressed in liver vascular cells [12,16,17,18,19,20]. Cx26 and Cx43 were found in sinusoidal endothelial cells [13] and of all three pannexins, Panx1 was found in the mouse hepatocytes as a contributor of the inflammasome, a caspase-1-activating multiprotein complex [21].

Cx26 and Cx32 were downregulated in acute liver failure, hepatitis and cholestasis in mice, rats and humans, respectively. Unlike liver fibrosis and cirrhosis where Cx32 was downregulated in rats and humans, in mice with non-alcoholic steatohepatitis was up or downregulated. Upregulation of Cx43 was observed in all mouse or rat liver diseases mentioned above, with no effect on expression in mice with non-alcoholic steatohepatitis. Panx1 was upregulated in mice with acute liver failure, hepatitis and cholestasis, as well as non-alcoholic steatohepatitis in humans and mice [12].

During gastrulation, the primitive gut tube is formed from the endoderm germ layer. The embryonic liver originates from the ventral foregut endoderm. At E9 hepatic diverticulum is formed and its anterior part gives rise to the liver and intrahepatic biliary tree, while the gall bladder and extrahepatic bile ducts derive from the posterior part. At E9.5, hepatoblasts form liver bud by delamination from the epithelium and invasion to the septum transversum mesenchyme (STM). From E10–15 the liver bud rapidly grows to become a major hematopoietic organ. Hepatoblasts are bipotential and differentiate mostly into hepatocytes, but those residing next to the portal veins become biliary epithelial cells. This process begins at E13 and continues until after birth. Around E8.5 in mice, hepatoblasts already express liver-specific proteins, like a-fetoprotein (Afp), hepatocyte nuclear factor (Hnf)4α, albumin (Alb) and transcription factors: hematopoietically-expressed homeobox (Hhex) and Prospero Homeobox 1 (Prox1). Contrary to hepatocytes, stromal cells, Kupfer cells, stellate cells and blood vessels originate from embryonic mesoderm [22,23].

An extracellular matrix glycoprotein reelin, in mammalian brain development, plays a role in prenatal neuronal cell migration, as well as postnatal neurotransmission, memory formation and synaptic plasticity [24]. Phosphorylation of the Disabled-1 (Dab1) protein, an intracellular adaptor of reelin signaling, is required for neuronal migration, as demonstrated by the observation of Dab1 knock-out mice and the similarity of the expressed phenotype to the *reeler* [25] (Reelin-/-) mice [26]. *Yotari*, an autosomal recessive mutant of the mouse *Dab1* gene, is recognizable by its premature death, unstable gait and tremor [27]. Previous findings are mostly based on neuronal abnormalities caused by *Dab1* deficiency, [28,29,30,31] but the role of the reelin signaling pathway in nonneuronal tissues and organs has not been studied until recently. Nowadays, multiple studies have addressed the role of reelin signaling in embryonic nonneuronal tissues such as the development of odontoblasts, small intestine, lymphatic vasculature, mammary gland, submandibular gland, cartilage and bone, as well as diseases and cancers in adulthood [32]. Furthermore, the appearance of Dab1 and reelin was demonstrated in human fetal kidney development [33] and possibly, Cxs and Panx1 could serve as markers of kidney function impairment [34] as it was shown in *yotari* mice [35].

Since Dab1 is not only brain-specific as was first thought, we want to examine the effect of *Dab1* deficiency on connexin and pannexin expression in mouse embryonic liver development.

## 2. Results

**Figure 1:**
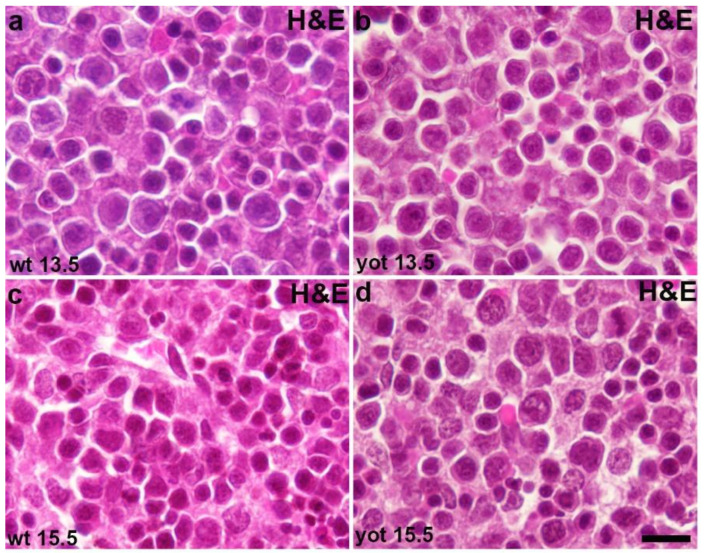
Hematoxylin-eosin (H&E) staining of the wt and *yotari* livers. H&E staining of the wt (**a**) and yot (**b**) livers at the embryonic day 13.5 (E13.5). H&E staining of the wt (**c**) and yot (**d**) livers at the embryonic day 15.5 (E15.5). The scale bar is 8 µm, refers to all images.

Cell to cell communication (i.e., gap junctions) disruption was examined by measuring immunoexpression of connexins (Cx26, Cx32, Cx37, Cx40, Cx43, Cx45) on mouse embryonic livers (*yotari* vs. wt) at a different stage of development (E13.5 and E15.5). The hemichannels formation, as well as propensity to apoptosis, was explored by measuring immunoexpression of pannexin 1 (Panx1) and apoptosis-inducing factor (AIF). Morphological differences between *yotari* and wt livers at E13.5 and E15.5 were observed by hematoxylin-eosin staining (Figure 1). Reelin expression was also examined to make an assumption about potential reelin signaling pathway in the liver, and ERK and mTOR expression were tested. As an inflammatory marker, TGF-β1 expression was examined.

### 2.1. Connexin (Cx) Expression

**Figure 2:**
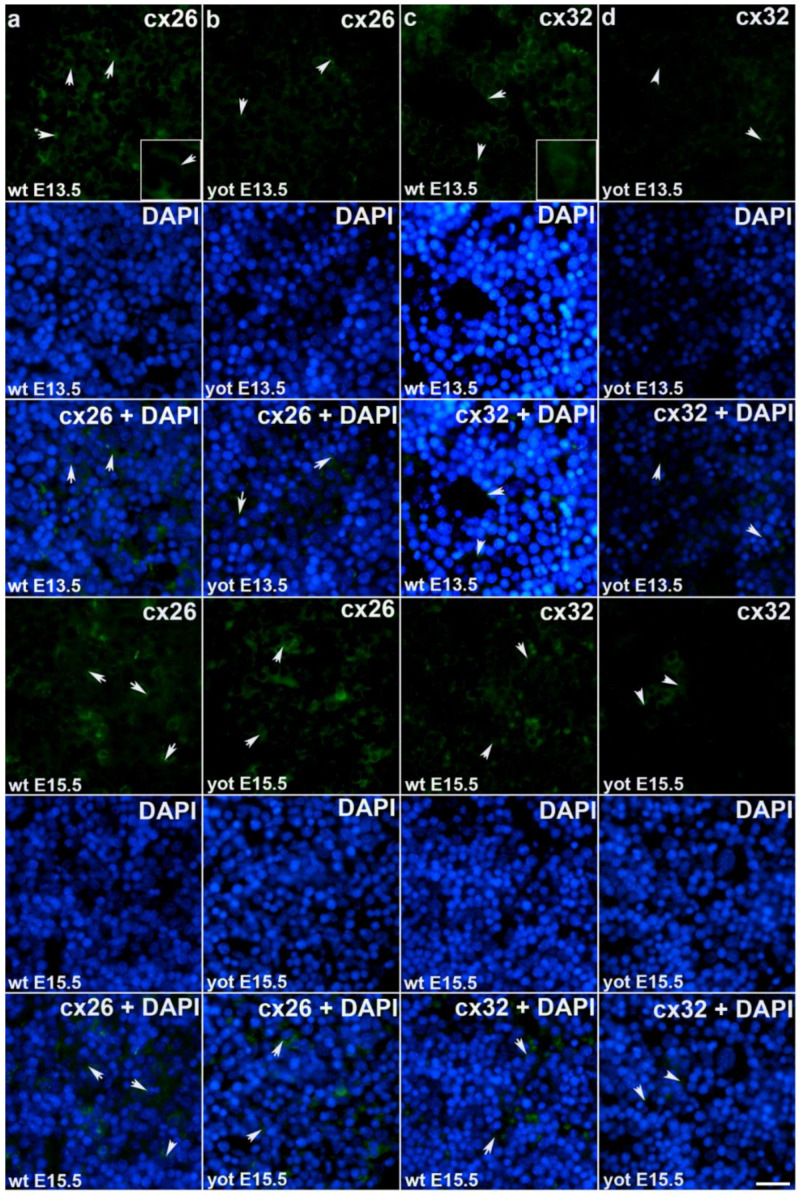
Immunofluorescence staining of connexins (Cxs): Cx26 and Cx32 in wild type and *yotari* mouse liver at gestation days E13.5 and E15.5. Immunoexpression of Cx26, 4′,6-diamidino-2- phenylindole dihydrochloride (DAPI) staining and merged Cx26 and DAPI at E13.5 and E15.5 in wild type (**a**) and *yotari* (**b**). Immunoexpression of Cx32, DAPI staining and merged Cx32 and DAPI at E13.5 and E15.5 in wild type (**c**) and *yotari* (**d**). * Autofluorescence of erythrocytes not to be mistaken with positive immunofluorescence staining of connexins. The scale bar is 8µm, refers to all images.

**Figure 3:**
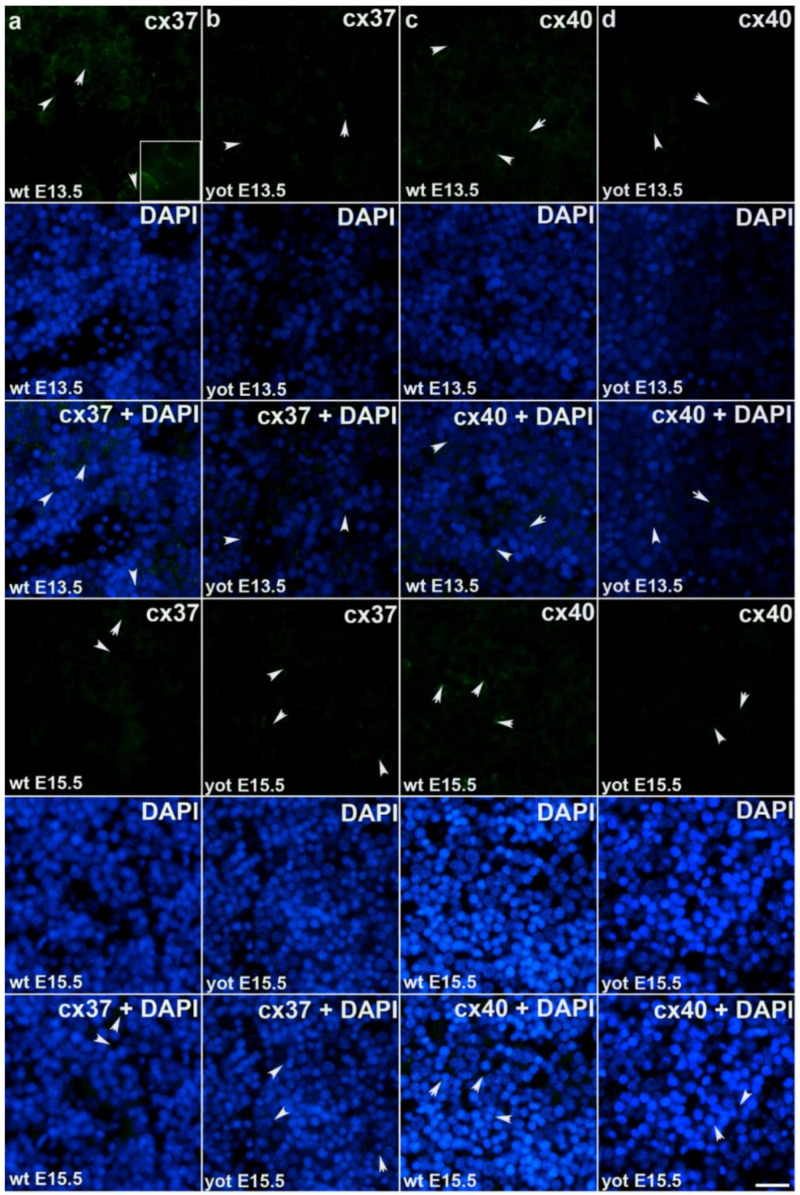
Immunofluorescence staining of connexins (Cxs): Cx37 and Cx40 in wild type and *yotari* mouse liver at gestation days E13.5 and E15.5. Immunoexpression of Cx37, 4′,6-diamidino-2- phenylindole dihydrochloride (DAPI) staining and merged Cx37 and DAPI at E13.5 and E15.5 in wild type (**a**) and *yotari* (**b**). Immunoexpression of Cx40, DAPI staining and merged Cx40 and DAPI at E13.5 and E15.5 in wild type (**c**) and *yotari* (**d**). The scale bar is 8 µm, refers to all images.

**Figure 4:**
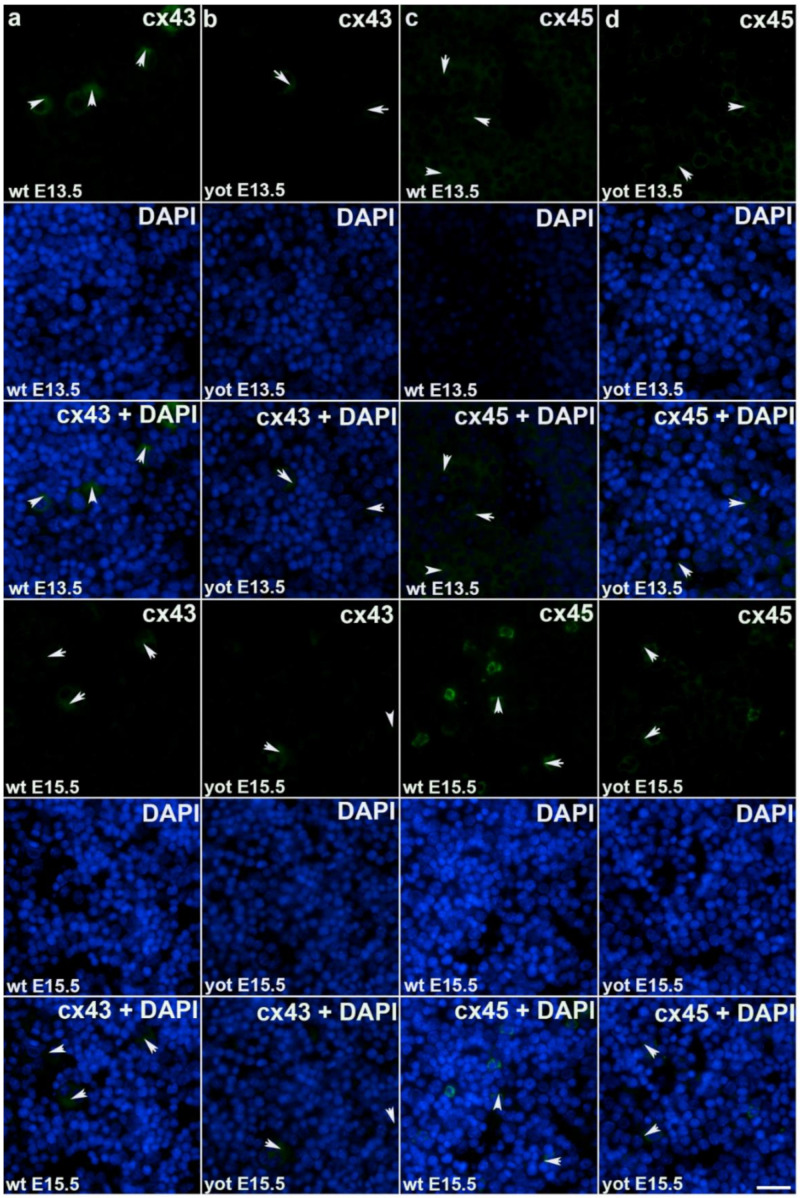
Immunofluorescence staining of connexins (Cxs): Cx43 and Cx45 in wild type and *yotari* mouse liver at gestation days E13.5 and E15.5. Immunoexpression of Cx43, 4′,6-diamidino-2–phenylindole dihydrochloride (DAPI) staining and merged Cx43 and DAPI at E13.5 and E15.5 in wild type (**a**) and *yotari* (**b**). Immunoexpression of Cx45, DAPI staining and merged Cx45 and DAPI at E13.5 and E15.5 in wild type (**c**) and *yotari* (**d**). The scale bar is 8 µm, refers to all images.

During early embryonic liver development (E13.5) all examined connexins (Cx26, Cx32, Cx37, Cx40, Cx43, Cx45) displayed expression in wild type (wt) mice in comparison to later developmental stage (E15.5) where lower values of expression were found with exception of Cx45 where wt mice showed significantly higher expression in later phase. Cx26 was equally expressed through embryonic stages E13.5 and E15.5 in wt individuals and it was strongly decreased in *yotari* embryos in both cases. The expression of Cx26 in *yotari* mice did not significantly differ between E13.5 and E15.5 (Figure 2a,b). Cx32 expression slightly decreased in E15.5 wt mice compare to E13.5, being significantly lower in *yotari* embryos at both developmental stages examined. The lowest expression was reached in *yotari* embryos at E15.5 (Figure 2c,d). Cx37 showed considerable expression in wt mice at E13.5, reaching the highest values of expression above all connexins explored. A notable decline (~5 fold) was observed for *yotari* mice at E13.5 in comparison to wt. There was almost no expression of Cx37 at E15.5, unlike *yotari* where an increase is detected. A mild reduction of Cx37 was noted between E13.5 and E15.5 *yotari* mice (Figure 3a,b). The expression of Cx40 in wt mice at E13.5 was remarkably dropping in *yotari* unlike wt, as well as wt and *yotari* at E15.5 where there was almost no expression (Figure 3c,d). Cx43 also showed low expression reaching the highest values in wt at E13.5. At the same phase, *yotari* demonstrated a significant decrease of Cx43 expression in contrast to wt. At E15.5 there was no significant difference between wt and *yotari*. Considering *yotari* at two different developmental phases there is a slight enhancement at E15.5 compared to the earlier phase (Figure 4a,b). Cx45 displayed a significant decrease in expression of *yotari* unlike wt in both embryonic stages monitored. A meaningful jump (2.6-fold) was observed in Cx45 expression at wt E15.5, compare to E13.5. No significance was found between the developmental phases of *yotari* (Figure 4c,d).

### 2.2. Pannexin1 (Panx1) and Apoptosis-Inducing Factor (AIF) Expression

**Figure 5:**
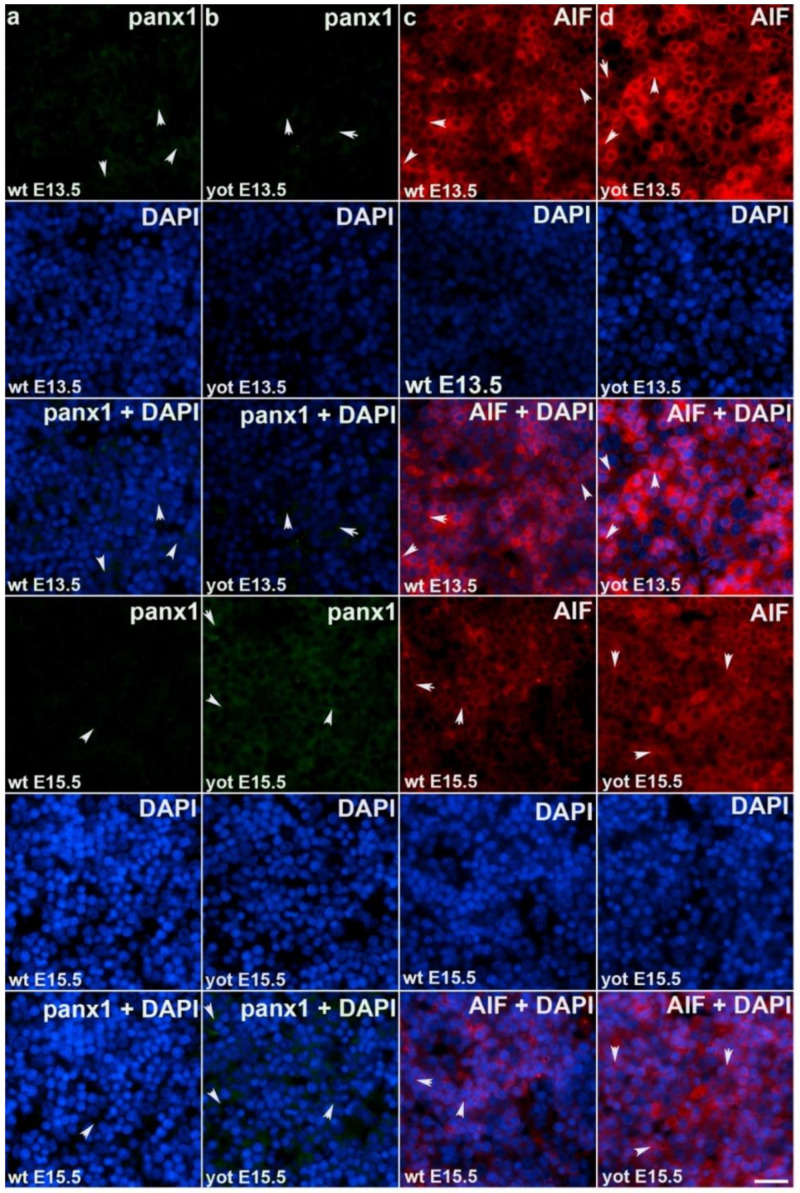
Immunofluorescence staining of pannexin1 (Panx1) and apoptosis-inducing factor (AIF) in wild type and *yotari* mouse liver at gestation days E13.5 and E15.5. Immunoexpression of Panx1, 4′,6-diamidino-2-phenylindole dihydrochloride (DAPI) staining and merged Panx1 and DAPI at E13.5 and E15.5 in wild type (**a**) and *yotari* (**b**). Immunoexpression of AIF, DAPI staining and merged AIF and DAPI at E13.5 and E15.5 in wild type (**c**) and yotari (**d**). The scale bar is 8 µm, refers to all images.

**Figure 6:**
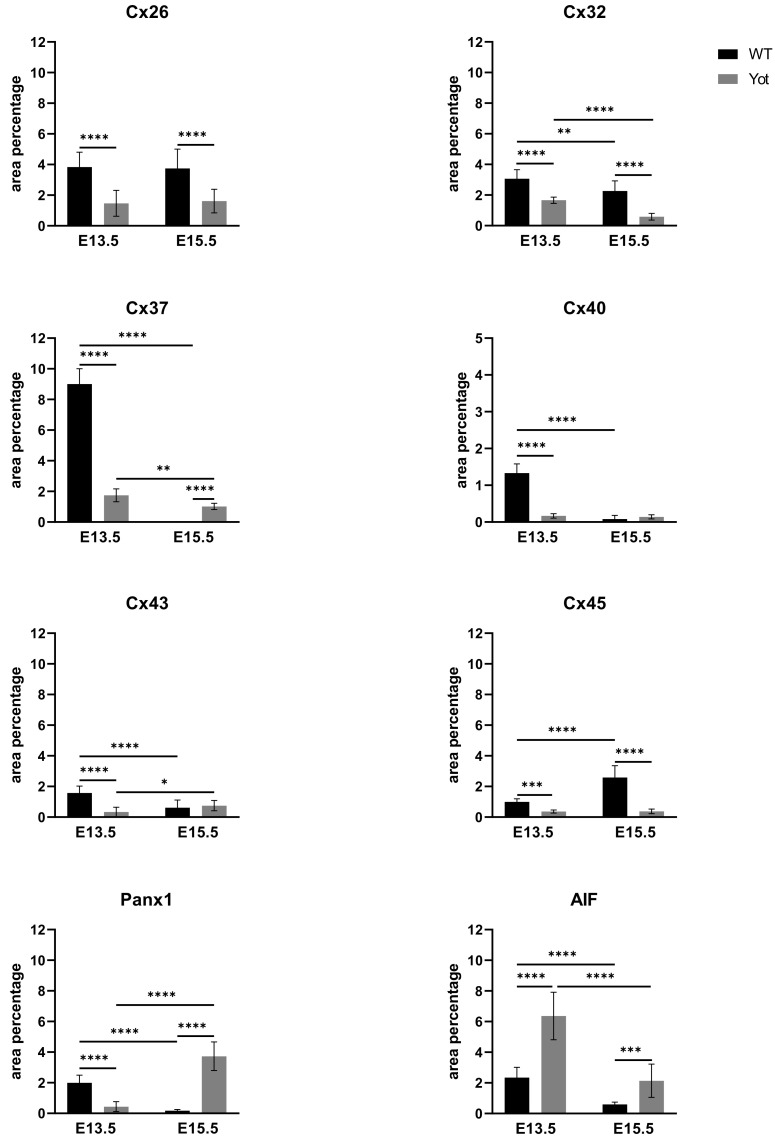
The area percentages of connexins (Cxs): Cx26, Cx32, Cx37, Cx40, Cx43, Cx45, pannexin1 (Panx1) and apoptosis-inducing factor (AIF) in wild type and *yotari* livers at gestation days E13.5 and E15.5. Data are presented as the mean ± SD (vertical line). Significant differences were indicated by * *p* < 0.05, ** *p* < 0.01, *** *p* < 0.001, **** *p* < 0.0001. One-way ANOVA followed by Tukey’s multiple comparisons test.

Observing Panx1 expression, opposite results were observed comparing wt and *yotari* at different stages of development. At E13.5, there was a decrease in Panx1 expression of *yotari* compared to wt, and later (E15.5) a strong increase (~21× fold) in expression was found. Also, if comparing the earlier and later phase of wt we have a significant reduction in expression while considering *yotari*, an increase in expression was present (Figure 5a,b and Figure 6). Looking at AIF expression significant increase was found in *yotari* compared to wt individuals in the observed phases. AIF expression is reduced in wt E15.5 compared to E13.5 as well as in *yotari* (Figure 5c,d and Figure 6).

### 2.3. Reelin (RELN), ERK, mTOR and TGF-β1 Expression

**Figure 7:**
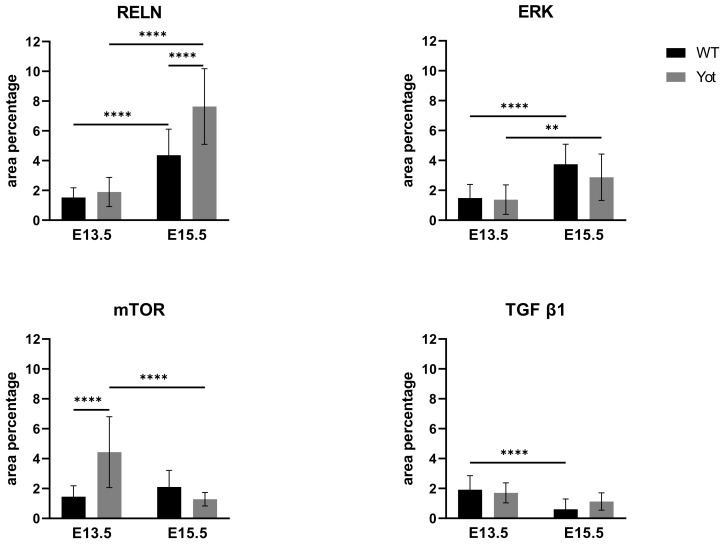
The area percentages of reelin (RELN), ERK, mTOR and TGF β in wild type and *yotari* livers at gestation days E13.5 and E15.5. Data are presented as the mean ± SD (vertical line). Significant differences were indicated by ** *p* < 0.01, **** *p* < 0.0001. One-way ANOVA followed by Tukey`s multiple comparisons test.

Looking at RELN expression only a significant increase was found at E15.5 yotari compared to wt (Figure 7 and Figure S1). ERK and mTOR expression didn’t follow this pathway since considering mTOR, only elevated expression is seen in *yotari* E13.5 and ERK didn`t display any significant difference between wt and *yotari* (Figure 7 and Figures S1 and S2). The same was shown questioning TGF-β1 expression between groups (Figure 7 and Figure S2).

## 3. Discussion

Knowing that liver connexin expression was altered upon liver organogenesis as well as in disease, we wanted to examine does Reelin signaling pathway also has some influence on mentioned using *yotari* (*Dab1-/-*) mice as a model organism. Even though the connexin expression was present in the preimplantation and during gastrulation, the exact mechanisms are still not well understood [36]. These transmembrane proteins, in form of gap junctions, enable passive intercellular diffusion of small (≤1 kDa) hydrophilic molecules such as cyclic adenosine monophosphate (cAMP), adenosine triphosphate (ATP), glucose, glutamate, glutathione, inositol triphosphate (IP3), ions like Ca^2+^, Na^+^, K^+,^ etc [37,38].

Our results indicate the importance of Cx26 and Cx32 expression in mouse liver development at E13.5 as well as E15.5, showing a significant decrease in *yotari* compared to wt. Even in the blastocyst stage of the mouse, Cx26 null mutation displays an embryonically lethal phenotype [36], unlike subtle alternations caused by Cx32 deficiency [39]. One-year-old mouse, deficient in Cx32 has a high incidence for spontaneous liver tumors [40]. A decrease of Cx26 and Cx32 production was demonstrated in hepatocellular carcinoma in humans [41,42]. Downregulation of Cx32 was also found upon the progression of alcoholic liver cirrhosis and chronic liver diseases. Acute liver injury, hepatitis and cholestasis [43] patients showed Cx26 and Cx32 decrease in expression, unlike Cx43 whose expression was increased [41]. The main gap junction protein of the liver, Cx32 is uniformly distributed while Cx26 is situated in the acinar periportal region, both being located in the plasma membrane of hepatocytes [42]. Since differentiation of hepatocytes and biliary epithelial cells from hepatoblasts start running around E13 our study doesn`t distinguish the location and cell type expressing mentioned proteins. The fact that these two are key liver connexins confirms not only their role in gap junction intercellular communication (GJIC) but their interaction with other proteins such as mitochondrial [44,45,46]. It should be further emphasized the role of connexins in hemichannels formation that are opened by the pathological trigger. It was shown that Cx32 hemichannels facilitate hepatocyte`s apoptotic to necrotic transition [46]. Many more reports describe connexin hemichannel`s role in tumorigenesis [47], death processes than their cytoprotection in cell survival [10]. Considering that we would also expect increased levels of connexins in *yotari* compared to wt which we didn`t get. It would be required to distinguish between gap junctions and hemichannels in reason of their different role. Using immunohistochemistry, we could not determine gap junctions or hemichannels in terms of connexins, in what regard we have studied pannexin expression to assume potential channel formation. At E15.5 we got a strong increase in expression of Panx1 of *yotari* compared to wt thereby confirming our hypothesis. To address the role of pannexin channels in apoptotic cells, Checkeni et al. explored nucleotide release in apoptotic cells using channel inhibitors in conjunction with exocytosis and membrane blebbing inhibitors to dispute the possibility of nucleotide release by the latter two. Accordingly, they pointed out the role of Panx1 in the release of ATP [48,49] and UTP from apoptotic cells using PANX1 knockdown and pharmacological inhibition carbenoxolone (CBX) targeting connexin and pannexin channels and probenecid, specific pannexin inhibitor [9]. Not to underestimate connexin hemichannel, both, connexin and pannexin (hemi)channels deserve further investigation to stress their role in physiological and pathological conditions as it was described previously [8]. Glycyrrhetinic acid and derivatives (e.g., CBX mentioned above), long-chain alcohols like heptanol and octanol, halogenated volatile anesthetics like halothane and enflurane, fatty acids of which some have antagonist role, fenamates, quinine and analogs, 2-aminoethoxydiphenyl borate (2-APB), polyamines and cyclodextrins, brilliant blue FCF, RNA based inhibitors (RNAi), antibody-based inhibitors (targeting the extracellular domain of Cxs but only in hemichannel form, not in gap junction where loops are occupied by docking interactions) and peptides based inhibitors were tested as potential therapeutics because of their ability to inhibit Cx and Panx channels [50]. There was also a possibility in our results in pannexin expression that we have an increase in E15.5 *yotari* because of delay in development compared to wt where Panx1 expression is present at E13.5. Our hypothesis that Panx1 could serve as a marker in liver pathology at the early stage of development concur with research showing that Panx1 knockdown reduces liver tumor metastasis in avian embryos [51]. Additionally, Panx1 facilitates inflammasome activation [41]. Looking at Cx37 expression we found strong expression in wt E13.5 whilst at E15.5 wt expression was negligible. Comparing wt and *yotari* we got weighty decreased expression at E13.5 contrary to E15.5 where *yotari* show a significant increase in expression, unlike wt. A decrease in Cx37 expression was found in most rat hepatic angiosarcomas, not because of mutation in the gene [52] what gives an assumption about its involvement in GJIC. Higher levels of Cx37 transcripts were found in mouse embryonic skin, brain and kidney unlike corresponding adult tissues [53] and our results indicate expression by hepatoblasts/hepatocytes et E13.5. There are a lot of reports demonstrating the role of Cx37 in vascular development, [54] likewise for Cx40 [55,56] and Cx43 [57,58] in agreement with our results where we got similar values in the embryonic liver, i.e., the highest level of expression is present at wt E13.5, a significant decrease in *yotari* at the same stage, almost no expression in wt E15.5, a significant increase (for Cx37) and negligible (for Cx40 and 43) in *yotari* E15.5. From the beginning of portal vein development (E11.5), Cx37 and Cx40 expression was observed in mouse livers, unlike hepatic veins and sinusoidal structures where expression was not found [16]. The perinatal death with vascular abnormalities was shown in Cx37-/- Cx40-/- mice. Cx37 deletion reduces Cx40 expression and vice versa, giving evidence of their codependency [56]. Liver rat progenitor cells express Cx43 and during oval cell differentiation into hepatocytes, there is a switch from Cx43 to Cx32 production. Furthermore, the highest levels of Cx43 transcripts were observed at E8-13 during oval proliferation and thereafter there is a reduction during their differentiation into hepatocytes [59]. This is in agreement with our results where we have highest expression in wt liver E13.5 and significant decline at E15.5. At the same time, looking at Cx32 expression our results display higher levels in both phases compare to Cx43. In more recent study, switch from Cx43 to Cx32 production was shown during hepatocytes differentiation of fetal hepatic progenitor cells from rats and moreover, inhibition of p38 MAPK pathway advance this process [60]. Contrary to our result where we got decline in Cx43 expression in *yotari* E13.5 compared to wt, in rodent liver overdosed with acetaminophen Cx43 is upregulated. Of note, colocalization with caspase 3 in rat liver drives at its role in cell death. Hepatocytes from Cx32-/- mice display reduced cell death upon acetaminophen treatment and Cx32-/- rodents show less liver damage compared to wt littermates. Along the same line, Cx43-/- mice showed increased cell death, oxidative stress and inflammation compared to wt littermates. This possibly indicates the role of Cx32 in spreading noxious messengers or in the removal of death cells as it was suggested by Crespo Yaguas et al. In hepatocarcinoma cells (HCC) decrease of Cx26 production is observed due to DNA methylation and Cx32 is localized in cytoplasm. Simultaneously Cx43 is present in cytoplasm and at the plasma membrane of HCC cells [41]. So far as we know, there are no reports pointing out the role of Cx45 in the liver. Our results indicate increase in Cx45 expression in wt E15.5 compared to wt E13.5. In *yotari*, both stages display decline compared to wt. Of note, gap junctions can be composed of the same or different type of connexins. In that view, scientists obtained that induction of Cx45 increases the amounts of Cx43 and Cx45 in rat liver epithelial cells [61]. Since we did not do colocalization of connexins we cannot confirm the possibility that Cx45 is part of heterotypic gap junctions what was shown in HeLa cells as well as in embryonic and adult mouse heart accentuating their electrophysiological properties [62,63]. Although morphologically there are no differences between *yotari* and wt livers, cell communication is disrupted according to our results. Apoptosis-inducing factor (AIF) is mitochondrial oxidoreductase that upon apoptosis and dissipation of mitochondrial membrane potential translocates from mitochondria to the nucleus in the caspase-independent manner [64]. Our results display significant increase in *yotari* livers compare to wt, and immunofluorescence results clearly show its cytoplasmic localization. It seems that in these developmental stages examined AIF shows higher expression levels in *yotari* what lead us to conclude that *yotari* are more prone to apoptosis. Since AIF is not translocated in the nuclei of *yotari* hepatoblasts/hepatocytes further study will be necessary to clarify this question.

Even though Dab1 protein was not detected within the adult liver, the liver was proposed as a place of reelin production [65]. On the contrary, more recent studies showed expression of Dab1 in hepatic progenitor cells of ductular reaction giving an assumption that reelin could promote fibrinogenesis through activation of hepatic stellate cells but also in restoring impaired hepatocytes. This study comes up with an idea of the paracrine role of reelin which was found upregulated in fibrosis. Taking into account that reelin activates human progenitor cells upon liver injury and the number of hepatic stellate cells increase as liver fibrosis progressed, reelin could serve as a potential candidate in liver pathology [66]. Another study demonstrated that reelin expression was restricted to stellate cells during early liver organogenesis in humans and rats respectively, but not in hepatocytes [67]. On the contrary, reelin expression was enhanced upon liver injury in hepatocytes and non-parenchymal cells as well [68]. In addition, along with developing sinusoids in the mouse embryo, reelin was detected in endothelial cells [69].

The main reelin signaling pathway for neuronal migration control includes apolipoprotein E receptor 2 (ApoER2) and very-low-density lipoprotein receptor (VLDLR), Src/Fyn kinases, Dab1 intracellular adaptor, and downstream Crk/Rap1 and adhesion molecules. Talking about reelin’s role in dendrite spine development, pathway goes through phosphoinositide 3-kinase/protein kinase/mammalian target of rapamycin (PI3K/Akt/mTOR) and reelin pathway responsible for learning and synaptic plasticity engages N-methyl D-aspartate receptor (NMDAR) as well as unidentified receptor (non-canonical pathway) for MEK/Erk1/2 pathway [26]. We checked mTOR expression in wt and *yotari* livers since reelin activates PI3K/Akt/mTOR, a pathway that is often hyperactivated in cancers. Also, we took into account ERK expression since that pathway possibly does not encompass Dab1. No significant differences were found between wt and *yotari* examining these proteins. The only mTOR showed an increase at E13.5 *yotari* compared to wt. PI3K/AKT, AMPK, Ras/Raf/MEK/ERK are just some of the upstream pathways that have been explored in liver diseases through the regulation of mTOR-mediated autophagy [70]. Thus, this increase in expression of mTOR at E13.5 *yotari* doesn’t answer a question about hyperactivation of the reelin signaling that implies mTOR. ERK could be part of the reelin signaling that does not engage Dab1 then drives through an unidentified receptor but also no differences were found in ERK expression.

Even though it is not enough known about reelin signaling in the liver, it had been demonstrated that blood reelin levels are significantly increased in patients with liver cirrhosis and fibrosis, and even more elevated in HCC patients [71]. Similar results were observed in another study where reelin upregulation was present in liver cirrhosis as well as its alteration in glycosylation in plasma from cirrhosis patients [72]. Our results showed elevated expression in *yotari* at E15.5 compared to wt so there is no doubt that reelin is involved in liver pathology, but the exact mechanism still needs to be clarified.

Transforming growth factor β1 (TGF-β1) in hepatocytes, during fibrosis, is responsible for the myofibroblast phenotype of hepatic stellate cells which then accumulate extracellular matrix proteins. TGF-β1 also mediates a process of epithelial-mesenchymal transition (EMT) in liver tumor cells that could increase the number of myofibroblasts [73]. Suppressed RELN expression has been observed in breast, pancreatic and gastric cancers. TGF-β1 is upstream of RELN and suppresses RELN expression, which results in increased cell migration ability of HCC [74]. Similar results were obtained in esophageal carcinoma cells, where RELN expression suppresses TGF-β1 induced migration [75]. TGF-β1 signaling plays role in intrahepatic bile duct development in which also alpha-smooth muscle actin (α-SMA) is expressed in the portal vein mesenchyme (PVM) adjacent to the bile ducts. Mutations in the TGFβ1 or its receptor in mice between E9.5 and E10.5 (before intrahepatic bile duct development) lead to lethality. At E10.5 TGF-β1 signaling if blocked, causes defects in bile ducts in the hilum portal veins and inhibits differentiation of biliary epithelial cells. From E14.5-18.5 α-SMA positive portal myofibroblasts were found in the portal vein mesenchyme adjacent to the biliary epithelial cells [76]. Since TGF-β1 is a known inflammatory marker and it is correlated with the reelin pathway we checked for its expression and didn`t find any significant difference between groups.

The limitation of this study is the lack of quantitative methods like Western blot or Elisa to confirm our immunofluorescence results.

In conclusion, our results emphasize connexins as a goal-keeper in the maintenance of liver homeostasis and Cxs and Panxs could serve as potential biomarkers and drug targets in liver pathology and diagnostics. We point out the role of Cxs and Panx during early liver development and possibly the role of the reelin signaling pathway as well. Further research will be required to stress the network between reelin signaling and GJIC. Considering that the reelin signaling pathway is taking place in liver injury it could affect transport through GJ in liver embryogenesis and other organs as well.

## 4. Materials and Methods

### 4.1. Animal Model and Processing

The C57BL/6N mice were housed in a temperature-controlled (23 ± 2 °C) environment with a 12 h dark/light cycle in polycarbonate cages with unrestricted access to laboratory chow and water. Previously described *yotari* mice [27,28] were used as Dab1 null conventional mutants. The following PCR primers were used for genotyping: *yotari*—GCCCTTCAGCATCACCATGCT and CAGTGAGTACATATTGTGTGAGTTCC, wild type—GCCCTTCAGCATCACCATGCT and CCTTGTTTCTTTGCTTTAAGGCTGT [69]. The gravid mice were sacrificed on 13.5 and 15.5 gestation days (E13.5 and E15.5) and their embryos were obtained. At first, they were anesthetized with pentobarbital and transcardially perfused using phosphate buffer saline (PBS, pH 7.2) and 4% paraformaldehyde (PFA) in 0.1 M PBS. After removal, embryos were separately fixed in 4% PFA in 0.1 M PBS overnight for conventional histological analyses: hematoxylin-eosin (H&E) and immunofluorescence (IF) staining.

### 4.2. Immunohistochemistry and Immunofluorescence

Following fixation, the tissue was dehydrated using graded ethanol solutions, embedded in paraffin blocks, sliced in 5 µm thick consecutive sections and mounted on microscopic slides. Every 10th section was stained with H&E staining for the verification of proper tissue preservation. After xylol deparaffinization and ethanol/distilled water rehydration, antigen retrieval was performed with sodium citrate buffer (pH 6) for 20 min at 95 °C in a water steamer and then progressively cooled down to room temperature and washed with phosphate buffer saline (PBS). To prevent nonspecific binding, protein blocking buffer (ab64226, Abcam, Cambridge, UK) was applied for 30 min at room temperature after which samples were stained with primary antibodies (Table 1) in a humidity chamber overnight. The following day samples were washed with PBS and suitable secondary antibodies (Table 1) were applied for one hour at room temperature protected from the light. Subsequently, slides were washed with PBS, stained with 40,6-diamidino2-phenylindole (DAPI) for nuclei detection, washed with distilled water and mounted on microscopic slides (Immuno-Mount, Thermo Shandon). No staining was observed when primary antibodies were excluded from the experimental procedure.

### 4.3. Data Acquisition and Statistical Analysis

Images were collected using a light microscope (for H&E tissue sections) and fluorescence microscope (Olympus BX51, Tokyo, Japan) equipped with a Nikon DS-Ri1 camera (Nikon Corporation, Tokyo, Japan). Fifteen representative visual fields of the mouse embryonic liver were captured with the same camera settings using 100× magnification applied with immersion oil (Carl Roth, Karlsruhe, Germany). Images were analyzed using ImageJ software (National Institutes of Health, Bethesda, MD, USA) and Adobe Photoshop (Adobe, San Jose, CA, USA). Green staining was considered as a positive Cx26, Cx32, Cx37, Cx40, Cx43, Cx45, Panx1, ERK, mTOR, TGF-β1 immunoexpression while red was interpreted as an apoptosis-inducing factor (AIF) and reelin (RELN) immunoexpression. Quantitative estimation of immunoreactivity was obtained using subtraction of the median filter and color thresholding to calculate the section percentage area covered by the positive signal. Statistical analyses were performed using GraphPad Software (GraphPad Software, La Jolla, CA, USA) with the probability level of *p* < 0.05 considered as statistically significant. One-way ANOVA test followed by post hoc Tukey’s test was carried out for comparison of immunoexpression of significant differences between groups. Images were analyzed by three researchers independently and three to four samples were used per group in each replicated experiment (*n* ≥ 3).

## Figures and Tables

**Table 1:** Primary and secondary antibodies used in the study.

	Antibodies	Catalog Number	Host	Dilution	Source
Primary	Cx26, GJB2	CSB-PA009452LA01HU	Rabbit	1:50	Cusabio (Wuhan, China)
	Cx32, GJB1	CSB-PA008853	Rabbit	1:100	Cusabio (Wuhan, China)
	Anti-Cx37/GJA4	ab181701	Rabbit	1:300	Abcam (Cambridge, UK)
	Anti-Cx40/GJA5	ab213688	Rabbit	1:50	Abcam (Cambridge, UK)
	Anti-Cx43/GJA1	ab87645	Goat	1:100	Abcam (Cambridge, UK)
	Anti-Cx45/GJA7	ab135474	Rabbit	1:50	Abcam (Cambridge, UK)
	Anti-pannexin1/PANX1	ABN242	Rabbit	1:150	MerckKGaA (Darmstadt, Germany)
	Human/Mouse/Rat AIF	AF5824	Sheep	1:300	R&DSystems (Minneapolis, MN, SUA)
	Anti-reelin E-5	sc-25346	Mouse	1:50	Santa Cruz Bt. (Texas, TX, SUA)
	P44/42 MAPK (Erk ½) 137F5	4695	Rabbit	1:250	Cell Signaling Techn. (Danvers, MA, USA)
	mTOR Polyclonal	PA5-34663	Rabbit	1:100	Thermo Fisher Scientific (Waltham, MA, SUA)
	Recombinant Anti-TGF beta1 EPR21143	ab215715	Rabbit	1:100	Abcam (Cambridge, UK)
Secondary	Donkey Anti-Goat IgG Alexa Fluor 488	ab150129	Donkey	1:400	Abcam (Cambridge, UK)
	Donkey Anti-Rabbit IgG Alexa Fluor 488	ab150073	Donkey	1:400	Abcam (Cambridge, UK)
	Rhodamine (TRITC) AffiniPure Donkey Anti-Rabbit IgG	711-025-152	Donkey	1:400	Jackson Immuno Research Laboratories, Inc., (Baltimore, PA, SUA)
	Rhodamine Red–XaffiniPure Donkex Anti-Sheep IgG	713-295-003	Donkey	1:400	Jackson Immuno Research Laboratories, Inc., (Baltimore, PA, SUA)
	Anti-mouse IgG Rhodamine Red	715-295-151	Donkey	1:400	Jackson Immuno Research Laboratories, Inc., (Baltimore, PA, SUA

## Data Availability

The data presented in this study are available on request from the corresponding author.

## Supplementary Materials

The following are available online at https://www.mdpi.com/article/10.3390/ijms221910712/s1.
