# Supplementary material for: Connexin Expression Is Altered in Liver Development of Yotari (dab1 -/-) Mice
Source: Int J Mol Sci. 2021 Oct 2;22(19):10712. doi: 10.3390/ijms221910712 (PMC8509723; doi:10.3390/ijms221910712)
Supplement: Supplementary file 1 [file ijms-22-10712-s001.zip › ijms-1380326-supplementary.pdf]

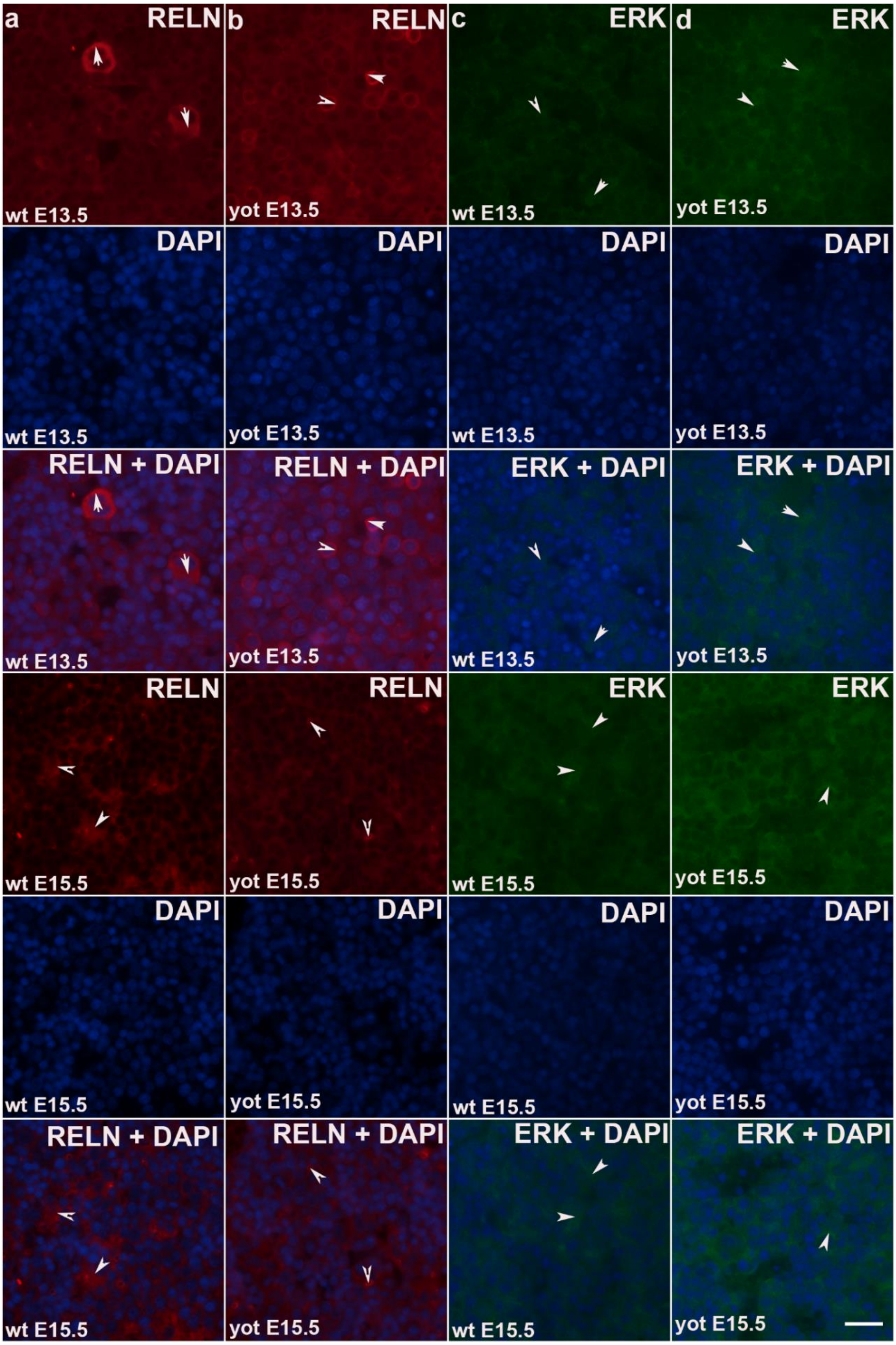

Figure S1. Immunofluorescence staining of reelin (RELN) and ERK in wild type and *yotari* mouse liver at gestation days E13.5 and E15.5. Immunoexpression of RELN, 4′,6-diamidino-2- phenylindole dihydrochloride (DAPI) staining and merged RELN and DAPI at E13.5 and E15.5 in wild type (**a**) and *yotari* (**b**). Immunoexpression of ERK, DAPI staining and merged ERK and DAPI at E13.5 and E15.5 in wild type (**c**) and *yotari* (**d**). The scale bar is 8um, refers to all images.

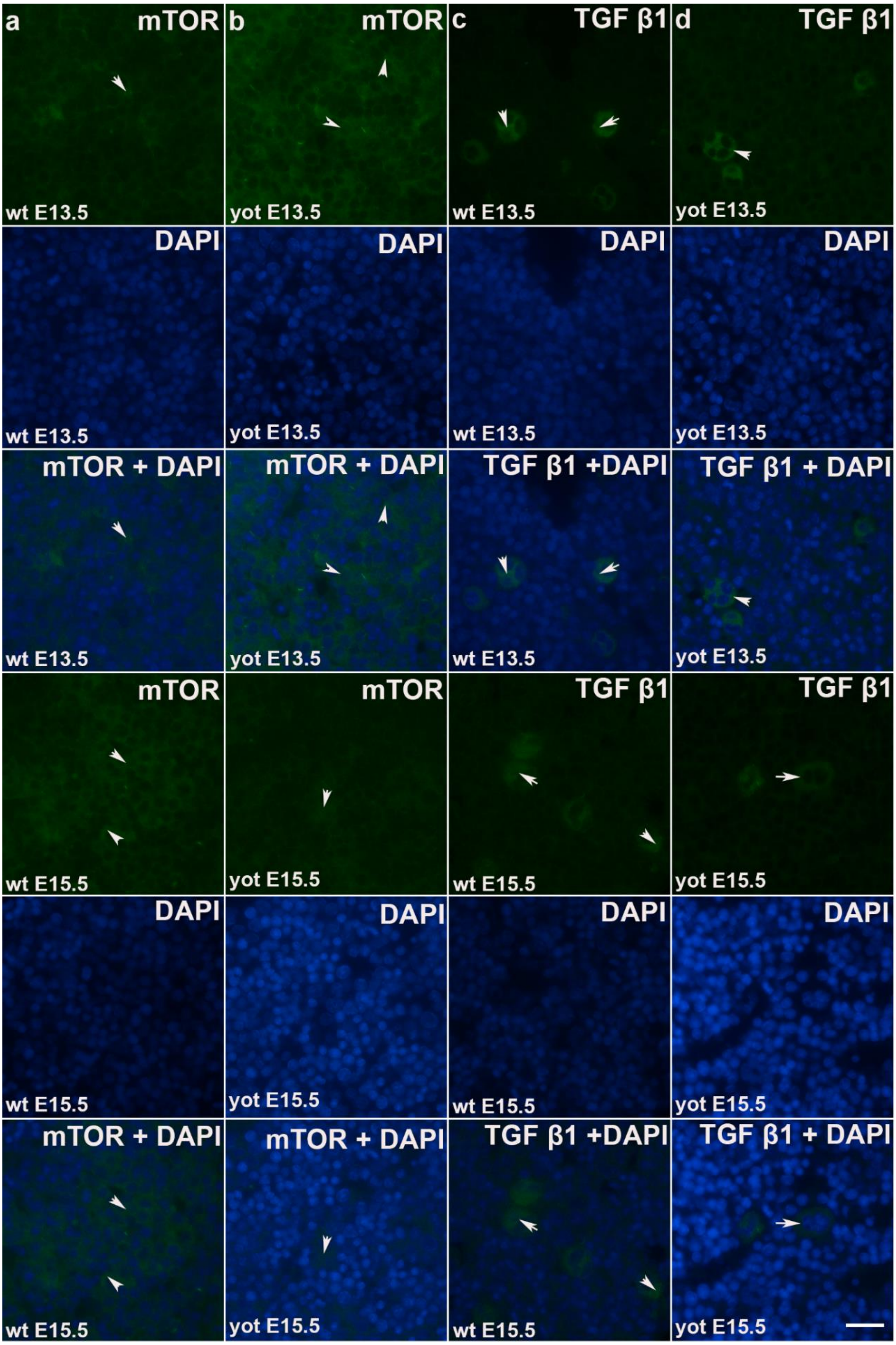

Figure S2. Immunofluorescence staining of mTOR and TGF β1 in wild type and *yotari* mouse liver at gestation days E13.5 and E15.5. Immunoexpression of mTOR, 4′,6-diamidino-2- phenylindole dihydrochloride (DAPI) staining and merged mTOR and DAPI at E13.5 and E15.5 in wild type (**a**) and *yotari* (**b**). Immunoexpression of TGF β1, DAPI staining and merged TGF β1 and DAPI at E13.5 and E15.5 in wild type (**c**) and *yotari* (**d**). The scale bar is 8um, refers to all images.
